# The Therapy of the Lactic Ferments

**Published:** 1908-02

**Authors:** 


### ABSTRACTS.

### MODERN THERAPEUTICS.

### The Therapy of the Lactic Ferments

THE most formidable intestinal bacteria are due to putrefaction. Bacteriological researches have shown that lactic ferments are the true enemies of putrefaction. The Bulgarian lactic ferments (fermenlactyl) in particular, are the most active to prevent it. Fermenlactyl tablets advantageously replace kephyr, yoghourt, and all other lactic ferments; and have no superior as an antiseptic for suppressing intestinal putrefaction

*Dyspepsia.*—Many dyspeptic troubles are due to abnormal fermentations causing pyrosis and a sensation of heaviness in the stomach. Acetic acids, butyric, etc., as well as chloric acid are found in the gastric juice. These acids are produced by microbian fermentations. Fermenlactyl destroys the bacteria and abolishes the uneasy sensations accompanying the digestion.

*Intestinal Troubles.*—Professor Pawlow, of St. Petersburg, has shown that the best stimulant for intestinal secretions is a solution containing an organic acid. Fermenlactyl realises this condition and it may be considered as the best intestinal tonic, stimulating as it does, the secretions of the liver and pancreas. Further, its lactic ferment destroys the noxious microbes of the intestines. In fetid diarrhea accompanying typhoid fever, enteritis, indigestion, etc., fermenlactyl is the nourishing diet that should be taken.

*Appendicitis.*—This disease is recognised as being of infectious origin, and as fermenlactyl combats intestinal infections, it is an excellent preservative and most easily supported nourishment.

*Diabetes.*—The lactic ferment contained in fermenlactyl destroys the sugar, and transforms it into matters inoffensive to our organism. Fermenlactyl is the nourishing element which will most rapidly diminish the quantity of glucose.

In conclusion fermenlactyl is indicated in the following diseases:

(*a*) Affections of the digestive tube, gastroenteritis, infantile diarrhea, enterocolitis, dysentery, intestinal tuberculosis, typhoid fever, chronic intestinal infection, and for the treatment of diabetes.

(*b*) Fermenlactyl is also indicated for reducing to a minimum the source of organic intoxication, cirrhosis of the liver, chronic nephritis, cardiac affections, gout, gravel, arteriosclerosis (old age), arthritis, furunculosis, urticaria, and the like.

(*c*) Fermenlactyl may also be prescribed in cases where patients are submitted to an alimentary diet liable to increase intestinal fermentation, such as the feeding of children with sterilised milk.

